# Polychaete (*Alitta virens*) meal inclusion as a dietary strategy for modulating gut microbiota of European seabass (*Dicentrarchus labrax*)

**DOI:** 10.3389/fimmu.2023.1266947

**Published:** 2023-12-11

**Authors:** Marta Monteiro, Simona Rimoldi, Rafaela S. Costa, Katerina Kousoulaki, Imam Hasan, Luisa M. P. Valente, Genciana Terova

**Affiliations:** ^1^ CIIMAR/CIMAR, Centro Interdisciplinar de Investigação Marinha e Ambiental, Universidade do Porto, Terminal de Cruzeiros do Porto de Leixões, Matosinhos, Portugal; ^2^ Department of Biotechnology and Life Sciences, University of Insubria, Varese, Italy; ^3^ Nofima, Nutrition and Feed Technology Department, Fyllingsdalen, Bergen, Norway; ^4^ ICBAS, Instituto de Ciências Biomédicas de Abel Salazar, Universidade do Porto, Porto, Portugal

**Keywords:** functional feeds, low-trophic organisms, aquaculture, gut microbiota, next generation sequencing

## Abstract

Recent research has revealed the significant impact of novel feed ingredients on fish gut microbiota, affecting both the immune status and digestive performance. As a result, analyzing the microbiota modulatory capabilities may be a useful method for assessing the potential functionality of novel ingredients. Therefore, this study aimed to evaluate the effects of dietary polychaete meal (PM) from *Alitta virens* on the autochthonous and allochthonous gut microbiota of European seabass (*Dicentrarchus labrax*). Two diets were compared: a control diet with 25% fishmeal (FM) and a diet replacing 40% of fishmeal with PM, in a 13-week feeding trial with juvenile fish (initial weight of 14.5 ± 1.0 g). The feed, digesta, and mucosa-associated microbial communities in fish intestines were analyzed using high-throughput sequencing of the 16S rRNA gene on the Illumina MiSeq platform. The results of feed microbiota analyses showed that the PM10 feed exhibited a higher microbial diversity than the FM diet. However, these feed-associated microbiota differences were not mirrored in the composition of digesta and mucosal communities. Regardless of the diet, the digesta samples consistently exhibited higher species richness and diversity than the mucosa samples. Overall, digesta samples were characterized by a higher abundance of Firmicutes in PM-fed fish. In contrast, at the gut mucosa level, the relative abundances of *Mycobacterium*, *Taeseokella* and *Clostridium* genera were lower in the group fed the PM10 diet. Significant differences in metabolic pathways were also observed between the FM and PM10 groups in both mucosa and digesta samples. In particular, the mucosal pathways of caffeine metabolism, phenylalanine metabolism, and sulfur relay system were significantly altered by PM inclusion. The same trend was observed in the digesta valine, leucine, and isoleucine degradation and secretion pathways. These findings highlight the potential of PM as an alternative functional ingredient in aquafeeds with microbiota modulatory properties that should be further explored in the future.

## 1 Introduction

Population growth and increasing demand for high-quality fish products pose a current challenge to the aquaculture sector, urging the identification of sustainable practices to meet food demand. The high dependence on fishmeal as the main protein source in aquafeeds has contributed to the overexploitation of marine resources and posed unbearable fluctuations in feed production costs (1, 2). In recent years, the adoption of good management practices has decreased the volume of unsustainable catches targeted for fishmeal production (from 30 Mt to 16 Mt) (3), resulting in a lower supply of marine-harvested ingredients for aquafeed production. Coupled with the rapid growth of the aquaculture industry, the limited supply of fishmeal has forced producers to rely on the use of alternative ingredients in the diets of farmed fish. This has led the feed industry to explore multiple alternative protein sources that often affect fish physiology and performance (4).

Since the suitability and sustainability of vegetable ingredients in diets for carnivorous fish have been increasingly questioned (5), other resources need to be explored, including ingredients that do not compete directly with the human food supply. Low-trophic marine species, such as polychaetes, have a well-balanced nutritional profile with high protein content and the ability to accumulate high levels of ω3 long-chain polyunsaturated fatty acids (LC-PUFA) and have shown potential to be included in aquafeeds (6–8). Moreover, marine polychaetes have been reported as a valuable source of bioactive compounds, including chitin and chitosan, which have been described as potential functional ingredients through their antimicrobial and anti-inflammatory actions and modulation of fish gut microbial communities (9).

A functional ingredient or additive refers to a feed component that goes beyond its nutrient effects and exerts physiological effects, thereby influencing one or more functions within an organism and contributing to improved performance, health, or enhanced disease resistance (10). These effects are frequently associated with changes in the gut bacterial communities (11). These communities are important in fish and contribute to host metabolism, nutrition, immune regulation, and disease resistance by preventing the colonization of opportunistic pathogens (12). Despite the lack of the knowledge on mammals and fish microbiota, over the last few years, many efforts have been made to bridge the gap (13–16) Research on fish microbiota has been strongly encouraged by the aquafeed industry, which has advantages in terms of new probiotics derived from the microbiota and the corresponding prebiotics. Coupled with the aim of reducing fishmeal incorporation in aquafeeds, various studies have demonstrated the impact of fishmeal replacement by other protein sources on the gut microbiota of fish (13, 17). For instance, a study on the partial replacement of fishmeal with a mix of poultry by-product meal and plant proteins indicated that the ratio between animal and vegetable proteins was a determinant of the gut microbiota profile in rainbow trout (*Oncorhynchus mykiss*). In particular, Firmicutes and Proteobacteria were discriminatory for diet type in fish with dietary plant ingredients, favoring a higher Firmicutes: Proteobacteria ratio than dietary land animal proteins (18, 19). Recently, low-trophic organisms have emerged as novel alternative ingredients in aquafeeds. For example, insects are characterized by high nutritional value, low production costs, and potentially lower environmental impact (20, 21). Their use in aquafeeds has yielded remarkable results as potential substitutes of fishmeal (22–31). with some remaining challenges to be met in terms of matching the performance of high quality fish meal (32). Insects contain a significant amount of chitin, which is an indigestible polysaccharide. It is considered a functional ingredient with a prebiotic action, which usually drives an increasing abundance of beneficial lactic acid bacteria and bacilli (17). In contrast, the impact of polychaetes as raw materials for aquafeeds on fish gut microbiota is yet to be explored. Therefore, there is no evidence for their possible modulatory effects on the gut microbial communities of cultured fish. Accordingly, this study aimed to assess the potential of polychaete meal (PM) obtained from *Alitta virens* as an alternative functional ingredient to replace 10% fishmeal in diets for European seabass diets by evaluating fish growth performance and gut microbiota.

## 2 Materials and methods

### 2.1 Ethical statement

The experimental trials in this study were performed by accredited scientists in laboratory animal science by the Portuguese Veterinary Authority (1005/92, DGAV-Portugal, following FELASA category C recommendations) and conducted according to Directive 2010/63/EU of the European Parliament and Council on the Protection of Animals for Scientific Purposes.

### 2.2 Ingredients and experimental diets

Two isoproteic (51% dry matter, DM), isolipidic (17% DM), and isoenergetic (22 kJ g^-1^ DM) diets were formulated and extruded by Nofima (Norway) with a pellet size of 2.0 mm. A control diet (FM) was formulated to contain 25% of fishmeal and compared to a diet containing 10% spray-dried PM (*Alitta virens*, Topsy Baits), at the expense of 40% fishmeal (PM10). The proximate composition of the ingredient and experimental diets is shown in **Table 1**. Further comprehensive characterization of these elements was performed in (33).

**Table 1:** Ingredients and proximate composition of the experimental diets.

Ingredient (%)	PM	FM	PM10
Fishmeal LT ^a^	-	25.0	15.0
Polychaete meal ^b^	-	0.0	10.0
Wheat gluten ^c^	-	5.04	6.04
Wheat ^d^	-	6.0	5.2
Fish Oil ^e^	-	9.2	10.0
Rapeseed oil ^f^	-	2.8	1.3
Soy Protein Concentrate ^g^	-	20.8	20.8
Horse beans ^h^	-	14.0	14.0
Corn gluten ^i^	-	7.50	7.50
Soybean meal ^j^	-	5.0	5.0
Rapeseed lecithin ^k^	-	0.5	0.5
Choline chloride^l^	-	0.5	0.5
Stay-C^l^	-	0.05	0.05
Vitamin mix ^l^	-	0.5	0.5
Mineral mix ^l^	-	0.5	0.5
NaH_2_PO_4_ ^l^	-	1.5	1.5
Lys (99%)^l^	-	0.50	0.55
Methionine (99%)^l^	-	0.20	0.25
Yttrium oxide^m^	-	0.01	0.01
H_2_O	-	0.40	0.80
Proximate composition			
Dry matter (%)	95.1	91.1	89.8
Ash (% DM)	16.4	8.9	8.5
Crude Protein (% DM)	65.5	51.8	51.0
Total Lipids (% DM)	10.9	16.6	16.6
Energy (kJ g^−1^ DM)	20.1	21.9	22.5
EPA+DHA (% DM)	0.8	1.3	1.3
Mineral composition (mg 100 g^-1^ DM)			
Phosphorus	-	1545.7	1346.1
Iron	-	28.7	29.2
Copper	-	1.8	1.9
Manganese	-	7.0	6.6
Zinc	-	18.8	20.2
Selenium	-	0.1	0.1

a Pelagia AS, Bergen, Norway.

b Raw material from Topsy baits, Wilhelminadorp, The Netherlands, Processed for the current trial by Nofima AS, Bergen, Norway.

c Tereos SYRAL Belgium N.V., Aalst, Belgium.

d Norgesmøllene AS, Bergen, Norge.

e Pelagia AS, Bergen, Norway.

f EMMELEV, Otterup, Denmark.

g CJ Selecta S.A., Araguari MG, Brazil.

h Soufflet, Grand Est, France.

i Roquette Frères, Lestrem, France.

j Fiskå Mølle, Etne, Norway.

k Berg + Schmidt, Hamburg, Germany.

l Vilomix Norway AS, Hønefoss, Norway.

m Vilomix Norway AS, Hønefoss, Norway.

### 2.3 Experimental conditions and feeding trial

Juvenile European seabass (*Dicentrarchus labrax*) were transported from Aquicultura Balear SAU, Spain to the Fish Culture Experimental Unit of CIIMAR, Portugal. For a 2-week period, the fish were acclimated to the experimental conditions while being fed a commercial diet (Aquasoja, Sorgal S.A.; 50% crude protein, 20% crude fat, in dry matter basis). Before the start of the trial, the fish were anesthetized and individually weighed (14.5 ± 1.0 g). Homogeneous groups of 40 fish were then distributed into 6 fiberglass tanks of 160-L in a saltwater re-circulation system. The system maintained appropriate levels of salinity (35‰), temperature (22.0 ± 0.5°C), dissolved oxygen, pH, and nitrogenous compounds throughout the trial, as recommended for this species (Blancheton, 2000). Each diet was randomly assigned to triplicate groups of fish, which were fed to apparent satiation three times daily (9, 12:30, and 16:30) using automatic feeders for 13 weeks.

### 2.4 Sample collection

At the end of the trial and 5 h after the last meal, all fish were lightly anesthetized for individual weighing (g). Then, six fish per tank were sacrificed using a sharp blow on the head for intestinal microbiota sampling. Before dissection, the external surface of each fish was wiped with 70% ethanol to avoid any accidental contamination from external body surface microbes. The intestine (excluding pyloric ceca) was aseptically removed with alcohol-disinfected instruments from each fish, and the digesta was collected by squeezing into sterile 2 mL tubes containing 800 μL RNAlater. The autochthonous intestinal bacteria were collected by scraping the intestinal mucosa with a sterile blade and placed in a sterile 2 mL tube with 300 μL of RNAlater. Both digesta and mucosa were pooled into three groups of two fish per tank (n = 9 per dietary treatment). Additionally, 10 g of each feed was collected for analysis of the feed microbiota. All the samples were stored at room temperature until DNA extraction.

### 2.5 Microbiota analysis

#### 2.5.1 Bacterial DNA extraction

The DNA extraction procedure has been described by (34). Briefly, DNA was extracted from 300 mg of digesta (nine samples/group), 200 µL of mucosal bacterial suspension (six samples/group), and 200 mg of each feed (three aliquots/feed). The DNeasy PowerSoil^®^ Pro Kit (Qiagen, Milan, Italy) was used for extraction according to the manufacturer’s instructions. The concentration and purity of DNA were spectrophotometrically measured using a NanoDrop™ 2000 spectrophotometer (Thermo Scientific, Milan, Italy). Bacterial DNA was stored at -20°C until NGS library preparation.

#### 2.5.2 Illumina NGS library preparation

The 16S amplicon sequencing library was prepared using the GalSeq srl sequencing service (Milan, Italy) according to the Illumina protocol “16S Metagenomic Sequencing Library Preparation for Illumina MiSeq System” (#15044223 rev. B). The composition of the microbial communities was determined by sequence analysis of the hypervariable region V4 16S rRNA gene, amplified using the primers 515F: 5′-GTGYCAGCMGCCGCGGTAA-3’ and 806R: 5′-GGACTACNVGGTWTCTAAT-3′. The libraries were sequenced on a NovaSeq 6000 System (Illumina) employing a paired-end 2 × 150 bp sequencing strategy with a cluster density of 300 K/sample. All raw read sequencing data were submitted to the public European Nucleotide Archive (EBI ENA), accession code: PRJEB61546.

#### 2.5.3 Raw sequencing data analysis

Raw sequencing data were analyzed using the QIIME 2 TM (version 2020.2) pipeline (35) and the taxonomic assignment of amplicon sequence variants (ASVs) was performed using the SILVA database (https://www.arb-silva.de/). The entire process of data elaboration included a pre-processing step, during which paired-end sequencing reads were adapter-trimmed, quality-filtered (Q > 30), and merged. The remaining high-quality reads were then dereplicated, and singletons and chimeric sequences were removed by running the qiime dada2 denoise-paired command. The output of the dada2 pipeline was an ASV table, which recorded the number of times each ASV was observed for each sample. Taxonomic classification was performed at the genus level. Eukaryotic, mitochondrial, and chloroplast sequences were removed. Alpha (within a single sample) and beta (between samples) diversity of bacterial communities were performed using QIIME alpha-phylogenetic and beta-phylogenetic commands, respectively.

For alpha diversity, the Chao 1, Faith PD, Observed ASVs, Shannon, and Simpson indices were calculated. For beta diversity, weighted and unweighted UniFrac distances were calculated depending on whether relative abundance or only presence/absence were considered. The UniFrac distances of individual samples were visualized using two-dimensional Principal Coordinate Analysis (PCoA) plots (36, 37).

#### 2.5.4 Predictive functional analysis of bacterial communities

PICRUSt (Phylogenetic Investigation of Communities by Reconstruction of Unobserved States) software package (38) was used to predict functional profiling of microbial communities using 16S rRNA marker gene sequences and the Greengenes (v.13.8) reference database. The entire data set of OTUs was used for the PICRUSt analysis. Metagenomic functions and pathways were predicted against KEGG pathways, as described in detail by Rimoldi et al. (27). The PICRUSt output files were then run on the Statistical Analysis of Metagenomic Profiles (STAMP) software package (39), generating extended error plots for each comparison.

### 2.6 Statistical analyses

Growth performance, feed intake, and feed alpha diversity data were analyzed using the Student’s *t*-test to assess statistically significant differences between dietary treatments. Differences were considered statistically significant at p < 0.05. Analyses were performed using SPSS, 21.0, IBM Corporation, 2011.

The alpha diversity data of the gut samples (digesta and mucosa) were analyzed by two-way ANOVA with diet and sample origin as independent factors. A non-parametric ANOSIM test with 999 permutations was applied to assess beta diversity dissimilarities among groups, whereas the remaining microbiota data (microbial relative abundances and metabolic pathways) were analyzed by Welch’s two-sided *t*-test using PAST3 software (40). In all comparisons, statistical significance was set at P < 0.05.

## 3 Results

### 3.1 Fish growth performance and feed utilization

**Figure 1:**
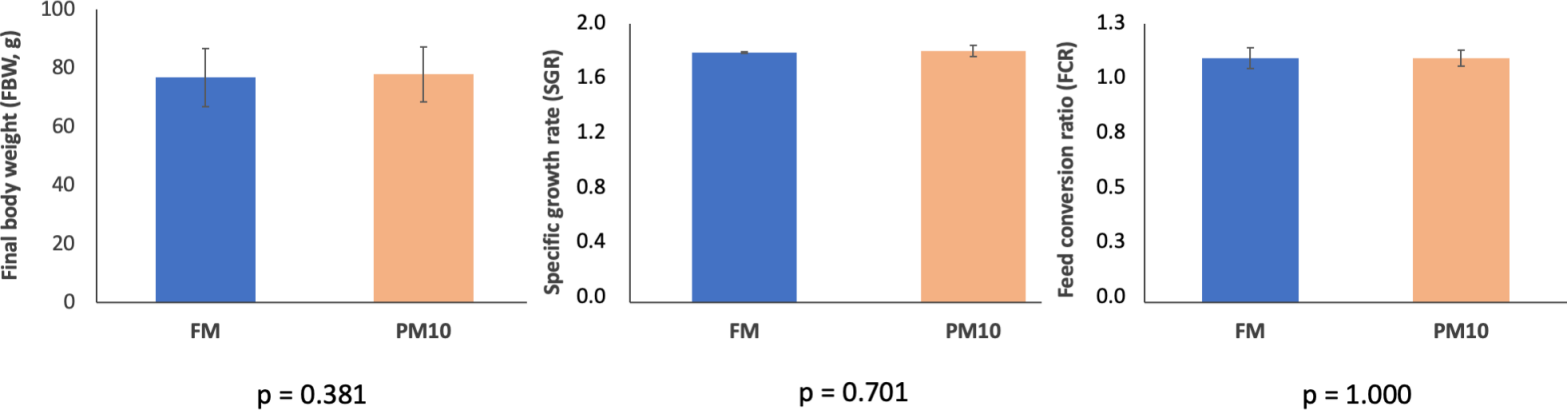
Growth performance and feed utilization of European sea bass fed the experimental diets. Values are expressed as mean ± SD (n = 3 tanks).

Both experimental diets were well accepted by European seabass juveniles, and mortality throughout the feeding trial was very low (< 1%). After 13 weeks, the fish quintupled their initial body weight, reaching an average of 77.9 g (**Figure 1**). Growth performance and feed utilization parameters, including SGR and FCR, were not significantly affected by the replacement of 40% of fishmeal with PM (**Figure 1**).

### 3.2 Microbiota analysis

#### 3.2.1 Sequencing efficiency

All feed and intestinal samples were efficiently sequenced, except for one mucosal sample from the PM10 feeding group that was discarded. A total of 2656796 high-quality sequences were obtained, specifically 479301, 1297769, and 879726 from the feed, digesta, and mucosa samples, respectively (**Supplementary Data File S1**). Good’s coverage value greater than 99% in all samples indicated that the bacterial communities were well representative. All sequencing data were submitted to the European Nucleotide Archive (EBI ENA) public database under accession code PRJEB61546.

#### 3.2.2 Alpha and beta diversity

Based on the rarefaction curves, the sequencing depth to calculate alpha diversity indices was set at 65172 reads. Alpha diversity analysis showed higher microbial biodiversity in the PM10 feed than in the FM feed, as indicated by the significantly higher values of Shannon and Simpson indices (**Table 2**). Concerning digesta or mucosa samples, no differences in species richness (Chao 1 and Observed ASV indices), and biodiversity (Shannon, Simpson, and Faith PD indices) were found in response to dietary treatments. However, regardless of the feed, species richness and biodiversity were higher in digesta samples than in mucosa samples (**Table 2**).

**Table 2:** Measurements of alpha diversity of gut and feed-associated microbiota.

	Chao1	Shannon	Simpson	FaithPD	Observed ASVs
Gut samples					
FM-DIG	1989 ± 302	7.74 ± 0.50	0.97 ± 0.01	21.65 ± 3.95	21.65 ± 3.95
PM10-DIG	2097 ± 591	7.33 ± 0.65	0.97 ± 0.01	23.15 ± 7.07	23.15 ± 7.07
FM-MUC	1621 ± 366	6.83 ± 0.39	0.96 ± 0.01	20.64 ± 5.56	20.64 ± 5.56
PM10-MUC	1364 ± 442	6.63 ± 0.36	0.96 ± 0.01	19.07 ± 6.75	19.07 ± 6.75
Two-way ANOVA					
Diet	0.817	0.208	0.349	0.951	0.725
Origin	**0.006**	**0.001**	**0.011**	0.316	**0.005**
Interaction	0.305	0.614	0.749	0.531	0.389
Feed samples					
FM-feed	1230 ± 69	6.16 ± 0.03^*^	0.95 ± 0.00^*^	17.98 ± 0.53	17.68 ± 0.53
PM10-feed	1252 ± 31	6.39 ± 0.01	0.96 ± 0.00	17.77 ± 0.36	17.77 ± 0.36

Data are expressed as mean ± SD. (*) p < 0.05. The significant p-values of two-way ANOVA are reported in bold. For t-test (*) indicates p< 0.05.

**Figure 2:**
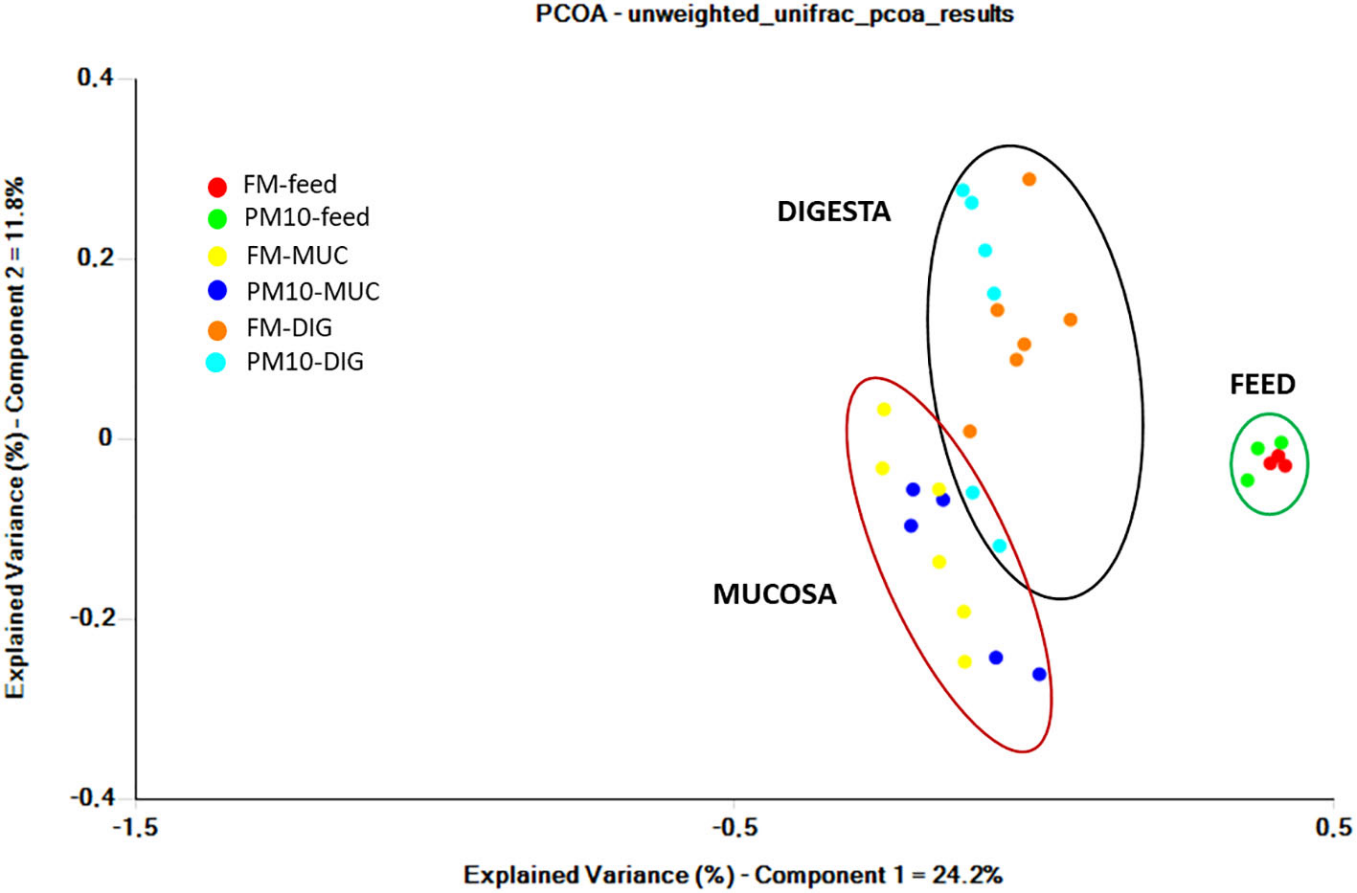
Principal coordinate analysis (PCoA) plot of unweighted UniFrac distances of gut microbial communities from gut (mucosa and digesta) and feed samples. Each point represents an individual.

Analysis of beta diversity showed significant differences among bacterial communities only for unweighted UniFrac metrics. The result of pairwise comparisons is reported in **Table 3**. The ANOSIM test revealed differences (p < 0.05) between the bacterial communities of the digesta and gut mucosa, regardless of the feed (**Table 3**). No difference was found between digesta samples from two dietary groups. Accordingly, the principal coordinate analysis (PCoA) plot of unweighted UniFrac distances of microbial communities revealed three separate clusters corresponding to the digesta, mucosa, or feed samples (**Figure 2**).

**Table 3:** Results of ANOSIM analysis of gut and feed samples based on Unweighted Unifrac distance values.

ANOSIM					
Group 1	Group 2	Sample size	Permutations	R	p-value
FM-feed	PM10-feed	6	999	0.07	0.309
FM-feed	FM-MUC	9	999	1.00	**0.014**
FM-feed	FM-DIG	9	999	0.95	**0.014**
PM10-feed	PM10-MUC	8	999	0.86	**0.022**
PM10-feed	PM10-DIG	9	999	0.91	**0.009**
FM-DIG	FM-MUC	12	999	0.60	**0.001**
FM-DIG	PM10-DIG	12	999	-0.04	0.651
FM-DIG	PM10-MUC	11	999	0.64	**0.002**
FM-MUC	PM10-DIG	12	999	0.28	**0.040**
FM-MUC	PM10-MUC	11	999	-0.11	0.869
PM10-DIG	PM10-MUC	11	999	0.456	**0.007**

Significant p –values (< 0.05) are in bold.

#### 3.2.3 Feed-associate bacterial communities

**Figure 3:**
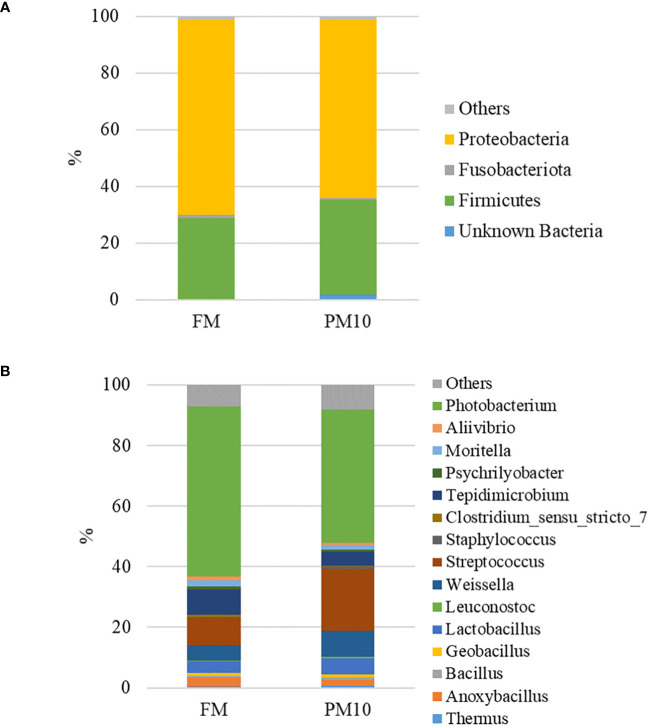
Feed-associated microbiota profiles at phylum **(A)** and genus **(B)** level. Mean relative abundance (%) of the most prevalent bacteria in FM and PM10 feed (N=3) is reported. Bacteria with relative abundance lower than 0.5% are pooled and indicated as “others”.

Considering only the most abundant taxa (>1%), the microbial profiles of the feeds were mainly comprised of three phyla (**Figure 3A**), five classes, eight orders, 12 families, and 15 genera (**Figure 3B**). Two sided Welch’s *t*-test analysis performed on the relative abundance data of bacterial taxa showed that 20 genera significantly differed between the two feeds. **Table 4** lists the genera displaying significantly different relative abundances, most of which belong to the Firmicutes phylum (11 out of 20). *Photobacterium* was the dominant genus in both feeds, and was significantly more abundant in FM than in PM feed. In contrast, genera belonging to the order Lactobacillales, represented by *Weisella*, *Lactobacillus*, *Streptococcus*, and *Lactococcus*, were more abundant in the PM10 than in the FM feed.

**Table 4:** List of bacterial feed-associated genera that significantly differed between two diets.

PHYLUM	CLASS	ORDER	FAMILY	GENUS	FM (%)	SD	PM (%)	SD	*Sig.*
Firmicutes	Clostridia	Clostridiales	Clostridiaceae	Clostridium_sensu_stricto_ 7	0.32	0.02	0.16	0.01	0.002
Firmicutes	Bacilli	Lactobacillales	Leuconostocaceae	Weissella	2.51	0.11	3.51	0.15	0.002
Proteobacteria	Gammaproteobacteria	Oceanospirillales	Endozoicomonadaceae	Endozoicomonas	0.00	0.00	0.46	0.04	0.004
Firmicutes	Bacilli	Lactobacillales	Lactobacillaceae	Lactobacillus	1.81	0.03	2.23	0.07	0.005
Firmicutes	Clostridia	Clostridiales	Clostridiaceae	Clostridium_sensu_stricto _5	0.06	0.01	0.01	0.00	0.005
Firmicutes	Bacilli	Bacillales	Planococcaceae	Kurthia	0.08	0.01	0.01	0.00	0.006
Fusobacteriota	Fusobacteriia	Fusobacteriales	Fusobacteriaceae	Fusobacterium	0.14	0.01	0.06	0.02	0.008
Proteobacteria	Gammaproteobacteria	Vibrionales	Vibrionaceae	Photobacterium	26.97	0.79	18.12	1.87	0.012
Firmicutes	Clostridia	Peptostreptococcales-Tissierellales	Peptostreptococcales-Tissierellales	Tepidimicrobium	4.02	0.08	1.90	0.39	0.014
Proteobacteria	Gammaproteobacteria	Alteromonadales	Moritellaceae	Moritella	1.02	0.11	0.58	0.05	0.014
Firmicutes	Bacilli	Lactobacillales	Streptococcaceae	Streptococcus	4.34	0.57	8.40	1.02	0.014
Proteobacteria	Gammaproteobacteria	Vibrionales	Vibrionaceae	Aliivibrio	0.53	0.04	0.31	0.01	0.015
Proteobacteria	Gammaproteobacteria	Burkholderiales	Neisseriaceae	Vitreoscilla	0.00	0.00	0.00	0.00	0.016
Proteobacteria	Gammaproteobacteria	Cardiobacteriales	Wohlfahrtiimonadaceae	Koukoulia	0.05	0.01	0.01	0.00	0.021
Firmicutes	Clostridia	Clostridiales	Clostridiaceae	Clostridium_sensu_stricto _1	0.24	0.03	0.13	0.02	0.022
Firmicutes	Bacilli	Bacillales	Bacillaceae	Anoxybacillus	1.43	0.19	0.76	0.09	0.023
Campilobacterota	Campylobacteria	Campylobacterales	frcobacteraceae	Pseudarcobacter	0.05	0.01	0.02	0.00	0.023
Firmicutes	Bacilli	Lactobacillales	Streptococcaceae	Lactococcus	0.06	0.00	0.11	0.01	0.025
Firmicutes	Bacilli	Lactobacillales	Vagococcaceae	Vagococcus	0.03	0.00	0.01	0.01	0.028
Proteobacteria	Gammaproteobacteria	Enterobacterales	Morganellaceae	Proteus	0.01	0.00	0.00	0.00	0.030

#### 3.2.4 Host-associate bacterial communities

**Figure 4:**
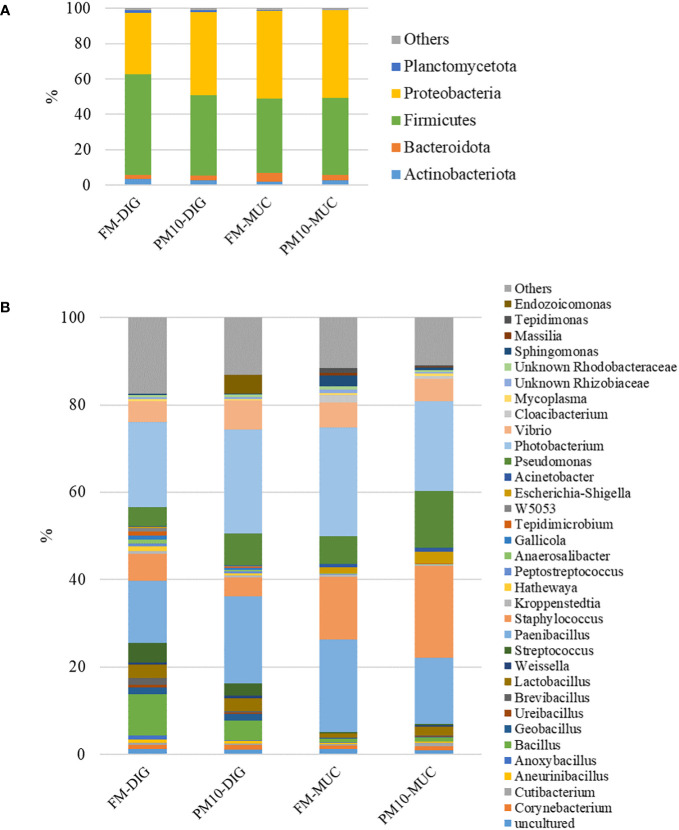
Gut microbiota profiles at phylum **(A)** and genus **(B)** level. Mean relative abundance (%) of the most prevalent bacteria in digesta (DIG) and mucosa (MUC) samples is reported (N=6). Bacteria with relative abundance lower than 0.5% are pooled and indicated as “others”.

Considering the entire set of intestinal samples (mucosa and digesta), the microbial profiles were mainly formed by five phyla (**Figure 4A**), seven classes, 20 orders, 29 families, and 31 genera (**Figure 4B**). Firmicutes and Proteobacteria constituted more than 90% of the total gut bacteria, followed by the Bacteroidota phylum, regardless of feed or sample type.

The results of the pairwise comparison between taxa relative abundance data from the samples showed a stronger effect of sample type (mucosa *vs.* digesta) than diet. Indeed, within the FM group, a total of six genera significantly differed between digesta and mucosa samples (**Table 5**), whereas for the PM10 feeding group, a total of 19 genera resulted in significantly different abundances between autochthonous and allochthonous gut microbiota samples (**Table 6**). In general, digesta samples were characterized by a higher abundance of Firmicutes, mainly represented by Lactobacillales, Bacillales, and Clostridiales orders (**Tables 5**, **6**). In digesta, the effect of diet was evident only in the *Anoxybacillus* genus, which was enriched in the FM feed group (**Table 7**). In contrast, at the gut mucosa level, diet affected the relative abundance of four genera: *Mycobacterium*, *Taeseokella*, *Clostridium_sensu_stricto* 1 and 7 (**Table 8**). All of these were more abundant in the FM diet group.

**Table 5:** List of genera in the control (FM) feeding group that significantly differed between mucosa and digesta samples.

PHYLUM	CLASS	ORDER	FAMILY	GENUS	DIG (%)	SD	MUC (%)	SD	*Sig.*
Firmicutes	Bacilli	Lactobacillales	Leuconostocaceae	Weissella	0.50	0.25	0.12	0.07	0.016
Firmicutes	Bacilli	Bacillales	Bacillaceae	Anoxybacillus	0.80	0.50	0.01	0.02	0.017
Actinobacteriota	Coriobacteriia	Coriobacteriales	Coriobacteriales_Incertae_Sedis	Raoultibacter	0.28	0.19	0.00	0.00	0.022
Firmicutes	Symbiobacteriia	Symbiobacteriales	Symbiobacteraceae	Symbiobacterium	0.06	0.04	0.00	0.00	0.022
Firmicutes	Clostridia	Peptostreptococcales-Tissierellales	Peptostreptococcales-Tissierellales	Gallicola	0.76	0.54	0.00	0.00	0.027
Firmicutes	Clostridia	Clostridiales	Clostridiaceae	Clostridium_sensu_stricto_18	0.31	0.18	0.09	0.03	0.037

**Table 6:** List of genera in the PM10 feeding group that significantly differed between mucosa and digesta samples.

PHYLUM	CLASS	ORDER	FAMILY	GENUS	DIG (%)	SD	MUC (%)	SD	*Sig.*
Firmicutes	Bacilli	Paenibacillales	Paenibacillaceae	Thermobacillus	0.04	0.01	0.00	0.00	0.003
Firmicutes	Clostridia	Lachnospirales	Lachnospiraceae	Lachnoclostridium	0.25	0.11	0.05	0.08	0.009
Firmicutes	Bacilli	Lactobacillales	Streptococcaceae	Streptococcus	2.55	1.37	0.27	0.33	0.013
Firmicutes	Clostridia	Peptostreptococcales-Tissierellales	Peptostreptococcaceae	Peptostreptococcus	0.37	0.21	0.02	0.04	0.014
Firmicutes	Clostridia	Clostridiales	Clostridiaceae	Clostridium_sensu_stricto_1	0.20	0.10	0.04	0.02	0.015
Actinobacteriota	Coriobacteriia	Coriobacteriales	Coriobacteriales_Incertae_Sedis	Raoultibacter	0.18	0.11	0.00	0.00	0.018
Firmicutes	Bacilli	Bacillales	Bacillaceae	Bacillus	4.13	2.36	0.65	0.38	0.021
Firmicutes	Bacilli	Lactobacillales	Leuconostocaceae	Leuconostoc	0.38	0.25	0.01	0.02	0.022
Firmicutes	Clostridia	Clostridiales	Clostridiaceae	Clostridium_sensu_stricto_18	0.18	0.09	0.04	0.07	0.023
Firmicutes	Bacilli	Staphylococcales	Staphylococcaceae	Staphylococcus	4.06	1.42	20.04	11.77	0.029
Firmicutes	Clostridia	Clostridiales	Clostridiaceae	Hathewaya	0.49	0.37	0.00	0.00	0.032
Firmicutes	Clostridia	Peptostreptococcales-Tissierellales	Peptostreptococcaceae	Clostridioides	0.13	0.08	0.02	0.03	0.035
Firmicutes	Bacilli	Lactobacillales	Enterococcaceae	Enterococcus	0.29	0.21	0.03	0.03	0.036
Firmicutes	Clostridia	Peptostreptococcales-Tissierellales	Peptostreptococcales-Tissierellales	Gallicola	0.36	0.28	0.00	0.01	0.037
Firmicutes	Thermovenabulia	Thermovenabulales	Thermovenabulales	Tepidanaerobacter	0.09	0.07	0.00	0.00	0.038
Firmicutes	Clostridia	Peptostreptococcales-Tissierellales	Peptostreptococcales-Tissierellales	W5053	0.13	0.11	0.00	0.00	0.039
Firmicutes	Clostridia	Clostridiales	Clostridiaceae	Clostridium_sensu_stricto_7	0.16	0.12	0.01	0.02	0.039
Firmicutes	Bacilli	Lactobacillales	Lactobacillaceae	Pediococcus	0.11	0.09	0.00	0.01	0.043
Firmicutes	Bacilli	Lactobacillales	Leuconostocaceae	Weissella	0.45	0.23	0.14	0.19	0.043

**Table 7:** List of genera that significantly differed in digesta samples between the two feeding groups.

PHYLUM	CLASS	ORDER	FAMILY	GENUS	FM-DIG (%)	SD	PM10-DIG (%)	SD	*Sig.*
Firmicutes	Bacilli	Bacillales	Bacillaceae	Anoxybacillus	0.80	0.50	0.21	0.18	0.045

**Table 8:** List of genera significantly differed in mucosa samples of two feeding groups.

PHYLUM	CLASS	ORDER	FAMILY	GENUS	FM-MUC (%)	SD	PM10-MUC (%)	SD	*Sig.*
Actinobacteriota	Actinobacteria	Corynebacteriales	Mycobacteriaceae	Mycobacterium	0.15	0.04	0.04	0.05	0.003
Bacteroidota	Bacteroidia	Cytophagales	Spirosomaceae	Taeseokella	0.27	0.08	0.07	0.10	0.007
Firmicutes	Clostridia	Clostridiales	Clostridiaceae	Clostridium_sensu_stricto_1	0.14	0.07	0.04	0.02	0.020
Firmicutes	Clostridia	Clostridiales	Clostridiaceae	Clostridium_sensu_stricto_7	0.14	0.10	0.01	0.02	0.031

#### 3.2.5 Predictive functional analysis of bacterial communities

**Figure 5:**
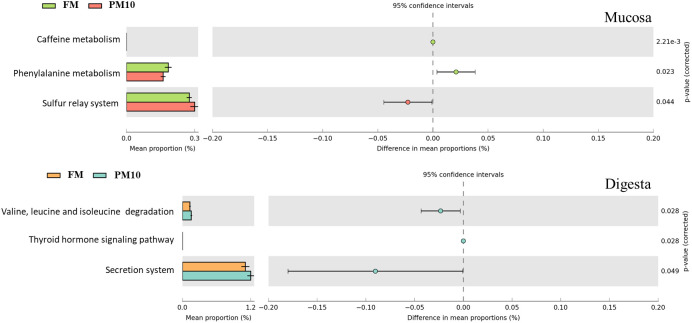
PICRUSt analysis results of predicted functional pathways in the gut microbiota.

To evaluate whether differences in microbial composition due to diet were associated with changes in microbial pathway expression, we performed PICRUSt analysis by comparing mucosa and digesta samples within the same feeding regime. This analysis showed a slight effect of diet on both mucosa and digesta. Only three pathways differed significantly between the FM and PM10 groups in both the mucosa and digesta. Specifically, caffeine metabolism, phenylalanine metabolism, and the sulfur relay system varied in the mucosa, whereas valine, leucine, thyroid hormone signaling, and isoleucine degradation and secretion system pathways varied in digesta samples from the two dietary treatments (**Figure 5**).

## 4 Discussion

In recent years, market forces driven by increased prices and reduced availability have led to the substitution of marine ingredients, including fishmeal, with plant-based alternatives (2, 41). However, this shift towards plant feedstuffs has recently been questioned because of sustainability concerns and a negative impact on fish immune response and stress resilience-associated alterations in the fish microbiome (5, 42, 43). This raises interest in exploring low-trophic organisms as fishmeal alternatives, especially since their incorporation into aquafeeds does not compete directly with the human food supply. This study demonstrated the potential of PM as an alternative ingredient in aquafeeds to replace up to 40% of high-quality FM without compromising European seabass growth and feed utilization.

Polychaete worms have been extensively used as a supplement in shrimp maturation diets (44), but only a few studies have explored the use of PM in fish diets. For instance, a study by Thum et al. (45) has shown that PM obtained from *A. virens* can be the main protein source in diets for rainbow trout, leading to similar growth performance to that of fish fed a commercial diet. Moreover, the inclusion of 40% PM dietary protein from *Nereis* sp. promoted the growth and survival of tilapia larvae (*Oreochromis niloticus*), supporting this ingredient as a suitable alternative protein source in aquafeeds (46). Furthermore, a recent study by Kals et al. (47) focused on the functional capabilities of PM derived from *Nereis virens* fed to the common sole (*Solea solea*). This study revealed that the ingestion of PM resulted in both enhanced growth and alleviation of anemia in these fish. These findings underscore the potential of using PM as a functional ingredient to promote growth and improve fish health. However, the specific mechanisms underlying these beneficial effects are currently unknown, warranting further investigation into the underlying mechanisms to fully comprehend its functional potential.

To our knowledge, this is the first study to evaluate the effects of partial dietary replacement of fishmeal with PM on the gut microbiota of cultured fish species. In the present study, 16S rRNA gene sequencing was successfully applied to characterize both gut mucosa-associated (autochthonous) and digesta (allochthonous) microbial communities in European seabass. Partial replacement of FM with PM did not have a marked effect on the gut microbial community profiles. Alpha diversity analysis revealed that neither species richness nor biodiversity of microbial communities in both mucosa and digesta were affected by the dietary inclusion of 10% PM. This is generally interpreted as a positive response of fish to dietary PM. The diversity of the gut microbiota is an indicator of good health. Fish diseases are often associated with dysbiosis and the loss of intestinal microbial diversity (48). In contrast to our previous findings in seabass (34), but in line with previous data in salmon (*Salmo salar*) (23), the transient microbiota was characterized by higher species richness and biodiversity than the resident microbiota, regardless of the dietary treatment. Similarly, beta diversity analysis showed substantial differences between digesta- and mucosa-associated intestinal microbiota, irrespective of diet, confirming what has already been reported in European seabass and other fish species (23, 28, 31, 34, 49). The transient and resident gut microbiota of European seabass was dominated by Proteobacteria, Firmicutes, and Actinobacteria phyla, which is consistent with existing literature (34, 50–52). However, if we compare the digesta- and mucosa-associated microbiota of fish fed the PM10 diet, the former was characterized by a higher abundance of Firmicutes. Specifically, an increased abundance of *Bacillus* and lactic acid bacteria (LAB), mainly *Pediococcus*, *Enterococcus*, *Leuconostoc*, *Streptococcus*, and *Weisella* genera, was found in digesta samples. Among these, *Leuconostoc*, *Enterococcus*, *Pediococcus*, and *Bacillus* genera are commonly used as probiotics in aquaculture (53). Interest in probiotics for animal production has been growing in recent years. In fact, studies have shown that they can improve fish growth and survival by improving the nutritional value of feed, and enhancing host disease resistance, respectively (54).

Considering the effect of the diet on gut microbial communities (digesta and mucosa), our data contradict previous findings on seabass and rainbow trout (28, 31, 34) and the general assumption that mucosa-associated intestinal microbiota is more resilient to dietary changes than digesta-associated microbiota. Specifically, we observed more pronounced changes in the mucosa-associated intestinal microbiota than in the digesta in response to the diet. At the resident microbiota level, partial substitution of FM with 10% PM reduced the relative abundance of the genera *Clostidium*_*sensu_stricto* 1 and 7. They are opportunistic pathogens that can cause intestinal inflammation in mammals (55, 56). *Clostridium* ss1 constitutes a large cluster of species that includes both commensal and pathogenic species. All members of *Clostridium* ss1 can ferment different carbohydrate substrates, producing butyrate as the final product (8). Interestingly, an interaction effect of diet and genotype was only found for *Clostridium sensu stricto* in selected for growth sea bass fed a “future” diet with low marine ingredients (50). However, in our study, we did not observe any advantage in terms of growth in the control FM group because of the higher abundance of this genus, probably due to the relatively rich diet of marine ingredients, fishmeal, and fish oil. At the digesta level, PM only negatively affected the *Anoxybacillus* genus, comprising cellulose-decomposing bacteria that usually populate the gut of herbivorous species such as grass carp (*Ctenopharyngodon idellus*) (57). Usually, this genus is enriched in the intestines of fish fed cellulosic feed. Although the amount was higher in the fecal matter of FM fish, the relative abundance of the *Anoxybacillus* genus in both digesta sample groups was lower than 1%, which is why there was no effect on feed utilization. In addition, based on the gut microbial profiles, we can clearly state that although there was a large relative abundance of LAB in the PM10 diet, they failed to colonize/establish fish guts. For the same reason, we can also state that gut microbial communities were not simply a mirror of feed-associated bacteria. To avoid this drawback and fully unveil the response of intestinal microbiota to diet, it is usually recommended to perform concurrent profiling of digesta- and mucosa-associated intestinal microbiota, as performed in the present study, rather than addressing only one or a combination of both. In the present study, PICRUSt analysis was used to estimate the differential functional capabilities of mucosa and digesta communities as a function of diet. As expected, no major significant differences were detected between the samples. These findings align with our earlier results, demonstrating that incorporating PM up to 10% in European seabass diets fosters comparable growth and nutrient utilization to FM (33).

In conclusion, our data support the potential of PM as an alternative functional ingredient in aquafeeds with microbiota-modulatory properties. Dietary supplementation with 10% PM seems to compensate for the possible drawbacks of high levels of FM substitution. Indeed, the replacement of 40% of fishmeal did not have any effect on the growth performance parameters of fish. However, the study revealed that PM has the potential to slightly modulate both digesta and gut mucosal communities, making it a promising alternative functional ingredient for aquafeeds. This study has also contributed to improving the knowledge of emerging ingredients for aquafeeds as alternatives to traditional marine and plant-based feedstuffs, envisioning the sustainable development of aquaculture.

## Data availability statement

The datasets presented in this study can be found in online repositories. The names of the repository/repositories and accession number(s) can be found below: https://www.ebi.ac.uk/ena, PRJEB61546.

## Ethics statement

The animal study was approved by Órgão Responsável pelo Bem-Estar Animal (ORBEA) - CIIMAR and conducted according to Directive 2010/63/EU of the European Parliament and Council on the Protection of Animals for Scientific Purposes. The study was conducted in accordance with the local legislation and institutional requirements.

## Author contributions

MM: Data curation, Formal analysis, Investigation, Writing – original draft, Writing – review & editing. SR: Data curation, Formal analysis, Writing – original draft, Writing – review & editing. RC: Data curation, Investigation, Writing – review & editing. KK: Resources, Writing – review & editing. IH: Investigation, Writing – review & editing. LV: Conceptualization, Data curation, Funding acquisition, Methodology, Project administration, Supervision, Validation, Writing – review & editing. GT: Conceptualization, Funding acquisition, Methodology, Supervision, Validation, Writing – review & editing.
